# CD8-Targeted PET Imaging of Tumor-Infiltrating T Cells in Patients with Cancer: A Phase I First-in-Humans Study of ^89^Zr-Df-IAB22M2C, a Radiolabeled Anti-CD8 Minibody

**DOI:** 10.2967/jnumed.121.262485

**Published:** 2022-05

**Authors:** Michael D. Farwell, Raymond F. Gamache, Hasan Babazada, Matthew D. Hellmann, James J. Harding, Ron Korn, Alessandro Mascioni, William Le, Ian Wilson, Michael S. Gordon, Anna M. Wu, Gary A. Ulaner, Jedd D. Wolchok, Michael A. Postow, Neeta Pandit-Taskar

**Affiliations:** 1 Department of Radiology, Perelman School of Medicine, University of Pennsylvania, Philadelphia, Pennsylvania;; 2 Abramson Cancer Center, Perelman School of Medicine, University of Pennsylvania, Philadelphia, Pennsylvania;; 3 Parker Institute for Cancer Immunotherapy, Memorial Sloan Kettering Cancer Center, New York, New York;; 4 Department of Medicine, Memorial Sloan Kettering Cancer Center, New York, New York;; 5 Department of Medicine, Weill Cornell Medical College, New York, New York;; 6 Imaging Endpoints, Scottsdale, Arizona;; 7 ImaginAb, Inc., Inglewood, California;; 8 HonorHealth Research Institute, Scottsdale, Arizona;; 9 Department of Molecular Imaging and Therapy, Beckman Research Institute of the City of Hope, Duarte, California;; 10 Molecular Imaging and Therapy, Hoag Family Cancer Institute, Newport Beach, California;; 11 Human Oncology and Pathogenesis Program, Memorial Sloan Kettering Cancer Center, New York, New York;; 12 Department of Radiology, Memorial Sloan Kettering Cancer Center, New York, New York; and; 13 Department of Radiology, Weill Cornell Medical College, New York, New York

**Keywords:** ^89^Zr-Df-IAB22M2C, PET imaging, CD8+ T cell, minibody, immunotherapy

## Abstract

There is a need for in vivo diagnostic imaging probes that can noninvasively measure tumor-infiltrating CD8+ leukocytes. Such imaging probes could be used to predict early response to cancer immunotherapy, help select effective single or combination immunotherapies, and facilitate the development of new immunotherapies or immunotherapy combinations. This study was designed to optimize conditions for performing CD8 PET imaging with ^89^Zr-Df-IAB22M2C and determine whether CD8 PET imaging could provide a safe and effective noninvasive method of visualizing the whole-body biodistribution of CD8+ leukocytes. **Methods:** We conducted a phase 1 first-in-humans PET imaging study using an anti-CD8 radiolabeled minibody, ^89^Zr-Df-IAB22M2C, to detect whole-body and tumor CD8+ leukocyte distribution in patients with metastatic solid tumors. Patients received 111 MBq of ^89^Zr-Df-IAB22M2C followed by serial PET scanning over 5–7 d. A 2-stage design included a dose-escalation phase and a dose-expansion phase. Biodistribution, radiation dosimetry, and semiquantitative evaluation of ^89^Zr-Df-IAB22M2C uptake were performed in all patients. **Results:** Fifteen subjects with metastatic melanoma, non–small cell lung cancer, and hepatocellular carcinoma were enrolled. No drug-related adverse events or abnormal laboratory results were noted except for a transient increase in antidrug antibodies in 1 subject. ^89^Zr-Df-IAB22M2C accumulated in tumors and CD8-rich tissues (e.g., spleen, bone marrow, nodes), with maximum uptake at 24–48 h after injection and low background activity in CD8-poor tissues (e.g., muscle and lung). Radiotracer uptake in tumors was noted in 10 of 15 subjects, including 7 of 8 subjects on immunotherapy, 1 of 2 subjects on targeted therapy, and 2 of 5 treatment-naïve subjects. In 3 patients with advanced melanoma or hepatocellular carcinoma on immunotherapy, posttreatment CD8 PET/CT scans demonstrated increased ^89^Zr-Df-IAB22M2C uptake in tumor lesions, which correlated with response. **Conclusion:** CD8 PET imaging with ^89^Zr-Df-IAB22M2C is safe and has the potential to visualize the whole-body biodistribution of CD8+ leukocytes in tumors and reference tissues, and may predict early response to immunotherapy.

## Visual Abstract

**Figure:**
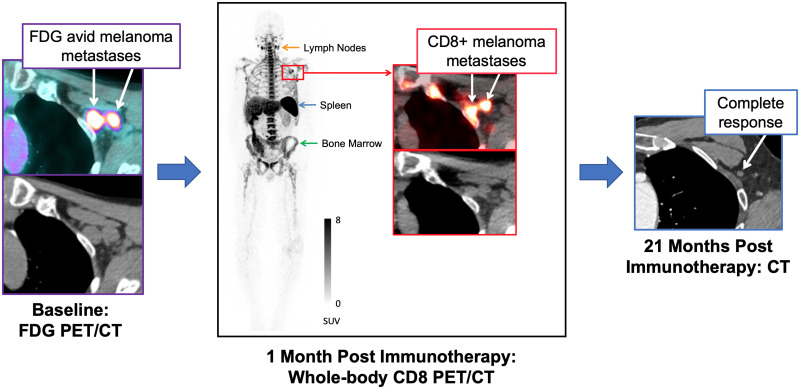

Immunotherapy has become standard of care for the treatment of many malignancies. Various strategies for enhancing the immune response to tumor antigens have been developed, most notably checkpoint inhibitors, as well as cancer vaccines, oncolytic viruses, and bispecific T-cell engager antibodies. In 2018, almost 44% of all cancer patients were eligible for treatment with checkpoint inhibitors based on U.S. Food and Drug Administration–approved regimens, but only a subset of patients respond (1–3).

T cells play a central role in the immune response to cancer, and tumor infiltration by CD8+ T cells, either on pretreatment biopsies or during the course of therapy, has been associated with response to immunotherapy (4–8). However, biopsies to assess T-cell infiltration are invasive and subject to sampling error, both within a lesion and across the entire burden of disease. Thus, a noninvasive method of visualizing CD8+ T-cell whole-body trafficking and tumor infiltration has the potential to play a pivotal role in guiding patient management by serving as an early measure of response, helping to select effective single or combination immunotherapies and facilitating the development of new immunotherapies by indicating pharmacodynamic activity. CD8 imaging may even play a role in identifying patients with tumors likely to be resistant to immunotherapy as well as in understanding immune-related adverse events resulting from immunotherapy.

IAB22M2C is a humanized 80-kDa minibody genetically engineered from the parent murine OKT8 antibody that targets human CD8 with high affinity. IAB22M2C is biologically inert, due to a lack of Fc receptor interaction domains, and has more rapid clearance than a full-sized antibody, giving it favorable properties for in vivo imaging. In vitro and in vivo preclinical studies with ^89^Zr-Df-IAB22M2C have shown that the probe does not impair CD8+ T-cell proliferation, activation, or cytotoxicity (9,10). In addition, preclinical PET imaging studies demonstrated the ability of ^89^Zr-Df-IAB22M2C to detect infiltrating CD8+ T cells in a variety of mouse models (9–11).

On the basis of these preclinical data, we initiated a first-in-humans study to evaluate ^89^Zr-Df-IAB22M2C in patients with solid tumors. An earlier report analyzed the data from the first 6 patients enrolled in the dose-escalation phase of the trial (12). Here, we report the results from the dose-expansion phase of the trial, which was designed to further explore minibody mass doses of the active pharmaceutical ingredient (API) for PET imaging and provide the final results of the safety, pharmacokinetics, biodistribution, and radiation dosimetry of ^89^Zr-Df-IAB22M2C in all patients enrolled in the phase 1 trial.

## MATERIALS AND METHODS

A prospective phase 1, open-label, nonrandomized, PET imaging study with ^89^Zr-Df-IAB22M2C was performed under an investigational new drug application (IND 127861). The protocol was approved by the Institutional Review Board, and all patients provided written informed consent (ClinicalTrials.gov identifier NCT03107663).

### Patients

Patients with histologically confirmed small cell or non–small cell lung cancer, squamous cell carcinoma of the head and neck, melanoma, Merkel cell carcinoma, renal cell carcinoma, bladder cancer, hepatocellular carcinoma, triple-negative breast cancer, gastroesophageal cancers, or Hodgkin lymphoma with at least 1 measurable lesion per RECIST 1.1 were eligible. Patients were either treatment-naïve or receiving standard-of-care therapy (without radiation therapy). All patients underwent baseline imaging, including CT or MRI performed as standard of care within 4 wk of ^89^Zr-Df-IAB22M2C administration. The study was conducted in 2 stages. During stage 1 of the trial, the total IAB22M2C mass dose was escalated, starting with 0.2 mg of API and increasing to 0.5, 1.0, 1.5, 5, and 10 mg of API consecutively for the first 6 patients. In stage 2 (dose-expansion), an additional 9 patients were randomly assigned to receive either 0.5 mg (*n* = 4) or 1.5 mg (*n* = 5), given the results from the dose-escalation cohort suggesting that lower minibody masses provided better visualization of CD8-rich tissues and tumor lesions (12). All patients underwent serial PET imaging for biodistribution and dosimetry analysis.

### ^89^Zr-Df-IAB22M2C Minibody Formulation

IAB22M2C minibody, obtained from ImaginAb, Inc., was conjugated to Good Manufacturing Practice–grade deferoxamine from Macrocyclics at the Radiochemistry and Molecular Imaging Core Facility at Memorial Sloan Kettering Cancer Center. Sterile Df-IAB22M2C was stored at 4°C for up to 2 wk before radiolabeling. ^89^Zr production and subsequent radiolabeling of Df-IAB22M2C were performed as previously described for other antibodies (13–15). Approximately 0.2–1 mg of Df-IAB22M2C was labeled with ^89^Zr and purified by a PD-10 column. The final product was supplemented with cold IAB22M2C minibody and diluted with formulation buffer, as needed. Before release, the final radiolabeled product was tested for appearance, pH, radiochemical identity, and purity by size-exclusion high-performance liquid chromatography and instant thin-layer-chromatography; for radionuclidic purity by γ-spectroscopy; for endotoxin level by portable test system reader; and for immunoreactivity by the bead method. Sterility testing was performed after release. The radiolabeling efficiency was >80%, radiochemical purity was >95% (as determined by instant thin-layer-chromatography), and minibody binding was >90%.

### ^89^Zr-Df-IAB22M2C Administration

A dose of 111 MBq (3 mCi) ± 20% of ^89^Zr-Df-IAB22M2C, in combination with cold IAB22M2C to make up the designated total mass balance, was administered intravenously over 5–10 min. No premedications were administered. Patients were monitored and vital signs measured for 1–2 h after injection, and also during additional imaging visits up to 48 h after injection. Electrocardiograms were recorded before and 10 min after injection. Side effects and reactions were graded per the Common Terminology Criteria for Adverse Events, version 4.0.

Blood samples were evaluated for antidrug antibodies (ADAs) at baseline, 3–4 wk after injection, and 8–12 wk after injection by BioAgilytix. Blood samples were evaluated for cytokines at baseline, and 4 and 24 h after injection by Charles River Laboratories.

### ^89^Zr-Df-IAB22M2C PET/CT Imaging and Analysis

Images were acquired at 3 centers using a Discovery 710 PET/CT scanner (GE Healthcare), a Discovery STE PET/CT scanner (GE Healthcare), or an Ingenuity PET/CT scanner (Phillips Medical Systems). Each patient underwent 4–5 whole-body PET/CT scans from the vertex of the skull to feet at 2–4, 24 ± 4, 48 ± 4, and 92–148 h after injection. If the patient agreed, an additional scan was acquired between the first and second scans at 6–8 h after injection. Emission scans were acquired in 3-dimensional mode at variable times per field of view (3 min on the day of injection, extending to 7 min at 92–148 h). PET/CT scans were acquired with low-dose CT for attenuation correction and lesion localization. A single low-dose CT scan at 24 h after injection was obtained with a 80 mA tube current (120 kVp; estimated radiation dose 9.0 mGy), whereas all other low-dose CT scans were obtained with a 10 mA current (120kVp; estimated radiation dose 1.1 mGy). Images were reconstructed with a 70-cm field of view into a 128 × 128 matrix using iterative ordered-subset expectation maximization (16 subsets; 2 iteration). All corrections recommended by the manufacturer were applied.

^89^Zr-Df-IAB22M2C PET/CT images were analyzed by Imaging Endpoints, LLC. Volumes of interest were drawn on PET/CT images over the lung, liver, spleen, kidney (left), muscle (paraspinal), aorta, bone marrow (L3 vertebrae), lymph nodes, and tumor lesions using dedicated software (mintLesion 3.2 software). All tumor lesions identified on baseline imaging studies were measured. For comparison of uptake trends, up to 3 target lesions per patient were analyzed; if more than 3 lesions were present, the largest lesions were selected. SUV was quantified using SUV_MEAN_ (normal tissues), SUV_PEAK_ (tumor lesions), or SUV_MAX_ (tumor lesions) normalized to lean body mass.

### Serum and Whole-Body Clearance Measurements

Multiple blood samples were obtained for assessment, including a baseline sample before ^89^Zr-Df-IAB22M2C infusion, followed by sampling at 5, 30, 60, 120, and 240 min after injection, and subsequently at the time of each PET scan, totaling 9–10 samples. Aliquots of serum were analyzed for radioactivity using a NaI (TI) γ-well-type detector (Wallace Wizard 1480 automatic γ-counter; Perkin Elmer); measured activity concentrations were decay-corrected and converted to percentage injected dose per liter. Aliquots of serum were also analyzed for ^89^Zr-Df-IAB22M2C using a validated enzyme-linked immunosorbent assay method by Charles River Laboratories. Activity in the whole body was determined on the basis of whole-body PET scans.

A biexponential function was fitted to the serum data, and a monoexponential function was fitted to the whole-body data using GraphPad Prism (version 8.4.3; GraphPad Software Inc.). Biologic clearance rates and corresponding half-times were derived from the fitted curves.

### Normal-Organ (Tissue) Dosimetry

Radiation dosimetry analysis on all 15 patients was conducted by CDE Dosimetry Services, Inc. Volumes of interest were drawn on PET images for all organs, showing uptake above general body uptake, including heart, lung, liver, gallbladder, spleen, bone marrow, kidney, small intestine, large intestine, salivary gland, testis, and urinary bladder. Data modeling, estimation of normalized number of disintegrations, and production of dosimetry estimates were performed using the RADAR (RAdiation Dose Assessment Resource) method for internal dosimetry as implemented in the OLINDA/EXM (version 1.1) software (16). All of these methods, including the image quantification, were also in general concordance with the methodology and principles as presented in MIRD pamphlet no. 16 (*17*). The effective dose (ED) was determined using the methodology as described in International Commission of Radiological Protection (ICRP) publication 103 (*18*). Additional details for the dosimetry analysis are provided in the supplemental materials (supplemental materials are available at http://jnm.snmjournals.org).

### Statistical Analysis

For patient demographics, medians and ranges were used to summarize continuous variables and percentages were used to summarize categoric variables. GraphPad Prism (version 8.4.3; GraphPad Software Inc.) was used for all statistical analyses. The results are indicated as mean ± SD, and *P* values less than 0.05 were considered significant; some results are shown as medians and interquartile ranges.

## RESULTS

Fifteen patients were enrolled (Table 1); 6 patients were enrolled in the initial dose-escalation phase (12) followed by an additional 9 patients in the dose-expansion phase. In the dose-escalation phase, 1 patient was enrolled in each of the following API dose groups: 0.2, 0.5, 1, 1.5, 5, and 10 mg; in the dose-expansion phase, 4 patients were enrolled in the 0.5-mg API dose group and 5 patients enrolled in the 1.5-mg API dose group. At the time of imaging, 8 patients were on immunotherapy, 2 patients had discontinued prior treatment with last dose > 5 mo before imaging, 3 patients were treatment-naïve, and 2 patients were receiving targeted therapy. The mean injected activity was 106 MBq (2.87 mCi), with a range of 93–121 MBq (2.52–3.26 mCi). The minibody mass of the radiolabeled product was 0.12 mg for the 0.2-mg dose level; for other levels, the mean (±SD) mass was 0.34 (±0.02) mg.

**TABLE 1.** Patient Characteristics

Characteristic	All patients (*n* = 15)
Median age (y)	64 (range, 30–81)
Sex (*n*)	
Male	9 (60)
Female	6 (40)
Tumor type (*n*)	
Melanoma	8 (53)
Non–small cell lung carcinoma	6 (40)
Hepatocellular carcinoma	1 (7)
Treatment profile at the time of imaging (*n*)	
On immunotherapy (<2 mo)	3 (20)
On immunotherapy (>2 mo)	5 (33)
On targeted therapy (1–6 mo)	2 (13)
Discontinued prior treatment (>5 mo)	2 (13)
Treatment naïve	3 (20)

Data in parentheses are percentages unless otherwise indicated.

### Safety and Tolerability

Injections were well tolerated, with no infusion site reaction higher than grade 1 reported. No adverse events related to the study drug were observed. There were no clinically significant changes in vital signs, blood chemistry and hematology, blood cytokines, or electrocardiograms. ADA analysis demonstrated transient immunoreactivity to ^89^Zr-Df-IAB22M2C in 1 of 15 patients at 3–4 wk after infusion, which became undetectable by 8–12 wk after infusion and was unaccompanied by symptoms or laboratory abnormalities.

### Pharmacokinetics

**FIGURE 1.**
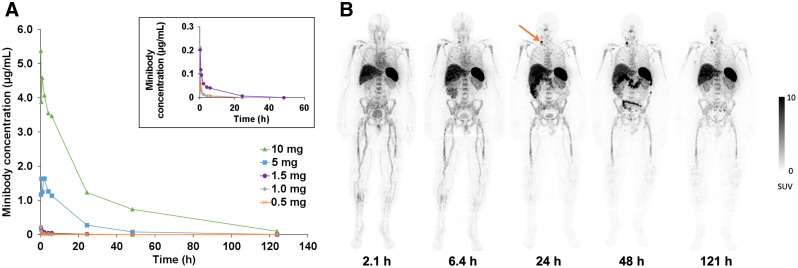
Serum clearance and biodistribution of ^89^Zr-Df-IAB22M2C. (A) Serum clearance of ^89^Zr-Df-IAB22M2C based on enzyme-linked immunosorbent assay measurements (limit of detection = 5 ng/mL). No minibody was detected in serum at the 0.2-mg dose. (B) Whole-body PET images of a patient at various times after injection of ^89^Zr-Df-IAB22M2C (1.5-mg minibody dose) demonstrating the distribution of ^89^Zr-Df-IAB22M2C in normal tissues and uptake in a nodal metastasis in the right neck (arrow), with good visualization of uptake in the nodal metastasis at 24–48 h after injection.

Serum clearance was biexponential and dependent on the mass of minibody administered, with more rapid clearance at lower masses (Fig. 1A) likely due to a greater proportion of target-mediated clearance. For the dose-expansion cohort in which patients received 0.5 or 1.5 mg of minibody, the biologic half-times were 0.33 ± 0.10 h (range, 0.17–0.46 h) for the fast component (α phase, 61.5%) and 14 ± 7.0 h (range, 2.7–25 h) for the slow component (β phase, 38.5%), based on serum radioactivity, and 0.38 ± 0.29 h (range, 0.12–1.1 h) for the fast component (α phase, 75.5%) and 6.4 ± 3.4 h (range, 0.83–11 h) for the slow component (β phase, 24.5%), respectively, based on enzyme-linked immunosorbent assay measurements of ^89^Zr-Df-IAB22M2C. At mass doses of 1.5 mg and lower, there was no detectable minibody in serum by 48 h after injection (Fig. 1A). Whole-body clearance for the dose-expansion cohort conformed to monoexponential kinetics, with a mean whole-body biologic half-life of 233 h (range, 71–341 h).

### Biodistribution and Normal-Tissue Uptake

In the dose-expansion cohort, ^89^Zr-Df-IAB22M2C cleared rapidly from the blood, with very low activity by 24 h after injection. The highest uptake was seen in the spleen, followed by bone marrow and liver (Fig. 1B). Liver uptake remained fairly constant over the imaging interval, whereas bone marrow and spleen uptake gradually decreased over time. The gallbladder had minimal to no uptake in most patients; in a few patients, the gallbladder was visualized at 2–6 h after injection, and cleared on later images. Uptake in the gastrointestinal tract was variable but generally peaked at 6–24 h and decreased thereafter, consistent with hepatobiliary clearance. Renal uptake was primarily cortical and increased over time, with similar activity compared with liver from 6 h after injection onward. Low-level activity was seen in the bladder in most patients at early time points, with minimal activity on later images.

**FIGURE 2.**
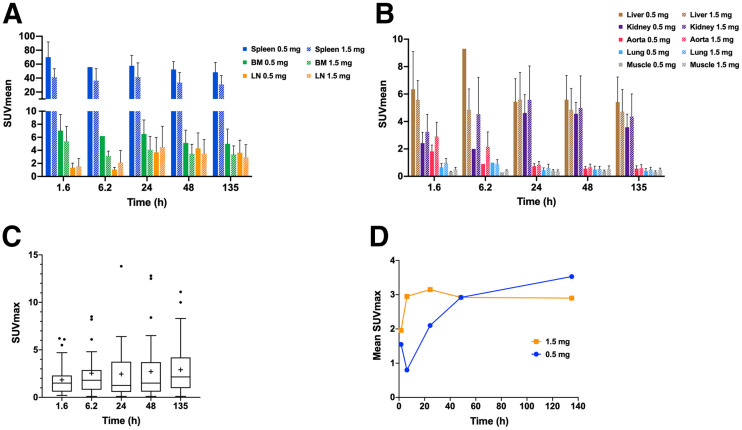
^89^Zr-Df-IAB22M2C uptake in normal tissues and tumor lesions versus time. (A) ^89^Zr-Df-IAB22M2C uptake in CD8-rich reference tissues in patients administered 0.5 and 1.5 mg of minibody mass. (B) ^89^Zr-Df-IAB22M2C uptake in CD8-poor reference tissues in patients administered 0.5 and 1.5 mg of minibody mass. (C) Box and whisker plots of ^89^Zr-Df-IAB22M2C uptake in tumor lesions from all subjects (*n* = 15). Boxes outline first and third quartile values. Median SUV_MAX_ values are indicated by horizontal line and mean SUV_MAX_ values are indicated with +. Outlier values are indicated by dots. (D) ^89^Zr-Df-IAB22M2C mean tumor uptake in patients who received 0.5 and 1.5 mg of minibody mass. BM = bone marrow; LN = lymph nodes.

^89^Zr-Df-IAB22M2C accumulated in CD8-rich tissues (e.g., spleen, bone marrow, and lymph nodes), with maximum uptake at 24–48 h after injection (Fig. 2A) along with low background activity in CD8-poor tissues such as muscle and lung (Fig. 2B). Normal lymph nodes were ^89^Zr-Df-IAB22M2C–avid in all patients, primarily in the cervical, axillary, and inguinal regions, but also in the mediastinum, hila, abdomen, and pelvis. Lymph nodes as small as 3 mm in short-axis diameter had an SUV_MAX_ of up to 6.9, and lymph nodes measuring 4 and 5 mm had an SUV_MAX_ of up to 11.8 and 17.4, respectively. Comparison of subjects in the dose-expansion cohort who were given 1.5 or 0.5 mg of API demonstrated reduced uptake in bone marrow and spleen at 1.5 mg of API but similar uptake in lymph nodes (Fig. 2A). In CD8-poor tissues (e.g., muscle and lung), no differences in uptake were noted between the 1.5- and 0.5-mg groups.

### Normal-Tissue Dosimetry

The average absorbed dose estimates for normal tissues are provided in Supplemental Table 1. The organs receiving the largest dose were the spleen at 12 ± 4.9 mGy/MBq followed by the kidneys at 2.3 ± 0.62 mGy/MBq and liver at 1.9 ± 0.50 mGy/MBq. The mean ED (effective dose, ICRP 103 (*18*)) was 0.65 ± 0.080 mSv/MBq. Comparison of groups in the dose-expansion cohort revealed similar dosimetry in subjects who received 1.5 mg of minibody compared with 0.5 mg, with a trend toward lower absorbed doses in the spleen (11 vs. 15 mGy/MBq, respectively) and bone marrow (0.68 vs. 0.81 mGy/MBq, respectively) and a lower mean ED (0.64 vs. 0.67 mSv/MBq, respectively) at the higher mass dose.

### Lesion Targeting and Uptake

**FIGURE 3.**
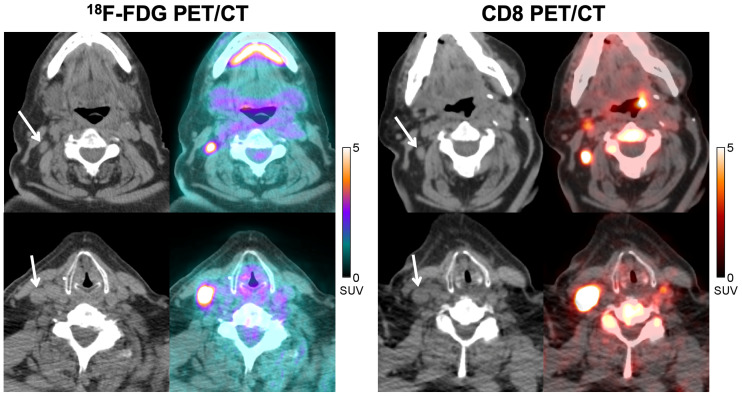
A 77-y-old man with metastatic melanoma treated with pembrolizumab. CT and fused ^18^F-FDG PET/CT images (left) acquired at approximately 8 mo after initiation of immunotherapy demonstrate 2 ^18^F-FDG–avid nodal metastases in right neck (SUV_MAX_ = 8.0, top image; SUV_MAX_ = 16.8, bottom image), which could represent viable metastases. Corresponding CT and fused CD8 PET/CT images (right) obtained at 1 mo after ^18^F-FDG PET/CT demonstrate significant tracer activity in both metastases (SUV_MAX_ = 5.4, top image; SUV_MAX_ = 14.6, bottom image), which suggests that some of the ^18^F-FDG activity could be due to tumor-infiltrating CD8+ T cells rather than tumor cells. Follow-up imaging over the next 6 mo demonstrated stable disease, supportive of this hypothesis.

Tumor lesion uptake data are listed in Supplemental Table 2. ^89^Zr-Df-IAB22M2C accumulated in tumor lesions, with maximum values 24–48 h after injection (Fig. 2C), similar to CD8-rich tissues. Radiotracer uptake in tumors was variable and noted in 10 of 15 (67%) patients, favoring slightly higher tumor uptake on average in the 1.5-mg cohort compared with the 0.5-mg cohort although this was not statistically significant (Fig. 2D). Tumor uptake above background was observed in 7 of 8 (88%) patients receiving immunotherapy, 1 of 2 (50%) patients who had discontinued therapy, 1 of 3 (33%) patients who were treatment-naïve, and 1 of 2 (50%) patients on targeted therapy. When ^89^Zr-Df-IAB22M2C uptake was analyzed by tumor type, the 2 largest cohorts (melanoma and non–small cell lung cancer) had similar ranges of tumor uptake with similar time–activity curves (results not shown). Several tumor lesions that were quite large had uptake at background (similar to blood pool), including metastatic lymph nodes measuring up to 5.4 cm and lung nodules measuring up to 4.7 cm (Supplemental Fig. 1). In addition, some tumor lesions that were small had significant uptake, such as a 0.7-cm metastatic lymph node with an SUV_MAX_ of 5.4 (Fig. 3).

**FIGURE 4.**
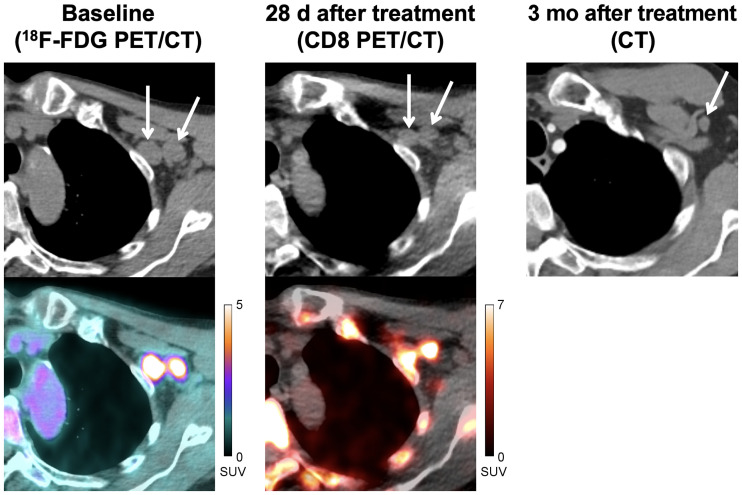
A 71-y-old man with locally advanced stage III melanoma treated with pembrolizumab. Baseline CT and fused ^18^F-FDG PET/CT images (left) demonstrate 2 ^18^F-FDG–avid metastases in left axilla (SUV_MAX_ = 10.0, medial node; SUV_MAX_ = 7.6, lateral node). CT and fused CD8 PET/CT images (middle) obtained at 28 d after start of immunotherapy demonstrate increased tracer activity in both metastases (SUV_MAX_ = 9.5, medial node; SUV_MAX_ = 10.0, lateral node), suggestive of tumor infiltration by CD8+ T cells. Follow-up imaging with contrast-enhanced CT (right) demonstrated complete response to therapy.

This trial was not designed to correlate tumor uptake with response to therapy; however, clinical follow-up was available for 3 patients. In 1 patient with regionally advanced melanoma, a CD8 PET/CT scan acquired 28 d after initiating immunotherapy (pembrolizumab) demonstrated marked ^89^Zr-Df-IAB22M2C uptake in 2 nodal metastases in the left axilla (SUV_MAX_ of 9.5 and 10.0) (Fig. 4), suggesting that the patient had a high degree of CD8+ leukocyte infiltration in the tumor; follow-up CT imaging in this patient demonstrated a complete response to therapy, which has lasted 2.3+ years. In another patient with metastatic melanoma, an ^18^F-FDG PET/CT acquired at approximately 8 mo after immunotherapy (pembrolizumab) initiation demonstrated ^18^F-FDG–avid metastases in the right neck with slightly increased size compared with prior studies that still qualified as stable disease. Subsequent CD8 PET/CT imaging, performed 1 mo after the ^18^F-FDG PET/CT, demonstrated marked ^89^Zr-Df-IAB22M2C activity in both metastases (SUV_MAX_ of 5.4 and 14.6) (Fig. 3), suggesting that the tumor had a high degree of CD8+ leukocyte infiltration; follow-up imaging over the next 6 mo supported the possibility this reflected a productive antitumor immune response because the patient experienced stable disease in these lymph nodes. In a third patient with metastatic hepatocellular carcinoma who progressed on sorafenib, CD8 PET/CT imaging performed 14 d after starting nivolumab demonstrated markedly increased ^89^Zr-Df-IAB22M2C activity in the primary tumor (SUV_MAX_ = 19.3) (Supplemental Fig. 2), suggestive of tumor infiltration by CD8+ leukocytes; follow-up CT imaging demonstrated a partial response to therapy, which has lasted 3+ y. In addition, the patient had an associated drop in α-fetoprotein from 33.2 ng/mL (pretreatment) to 1.4 ng/mL (3 y after initiation of therapy).

## DISCUSSION

A noninvasive method of visualizing CD8+ T-cell whole-body biodistribution and tumor infiltration, both before and during therapy, has the potential to play a pivotal role in guiding patient management. In this first-in-humans trial, CD8-targeted PET imaging with ^89^Zr-Df-IAB22M2C, a humanized anti-CD8 minibody, was demonstrated in patients with a variety of malignancies. An earlier report analyzed the data from the first 6 patients enrolled in the dose-escalation phase of the trial (12). Here, we report the final results from the trial, including results from the dose-expansion phase, which was designed to identify the optimal minibody mass dose for PET imaging. In this study, ^89^Zr-Df-IAB22M2C was found to be safe and well tolerated, with no infusion reactions higher than grade 1 and no drug-related adverse events. ADAs were detected in 1 patient at 3–4 wk after infusion, which became undetectable by 8–12 wk after infusion.

The biodistribution of ^89^Zr-Df-IAB22M2C was consistent with CD8+ leukocyte targeting: not all CD8+ leukocytes are T cells, with robust uptake of ^89^Zr-Df-IAB22M2C in CD8-rich tissues (e.g., spleen, bone marrow, and lymph nodes) with maximum uptake at 24–48 h after injection, and relatively low uptake in CD8-poor tissues (e.g., muscle and lung). Radiotracer-avid normal lymph nodes were frequently seen in the neck, axilla, and inguinal regions, which is expected, as these are common sites for reactive processes due to infectious or environmental stimuli. Even very small lymph nodes (measuring 3 mm in short-axis diameter) were radiotracer-avid, suggesting that the imaging probe has high sensitivity for CD8+ leukocytes. In addition, ^89^Zr-Df-IAB22M2C uptake in CD8-rich tissues was saturable, with lower uptake in the spleen and bone marrow in the 1.5-mg cohort than in the 0.5-mg cohort. No differences in lymph node uptake were seen between the 1.5- and 0.5-mg cohorts, possibly due to greater blood flow to, and availability of, target sites in the spleen and bone marrow relative to lymph nodes. In CD8-poor tissues (e.g., muscle and lung), no differences in uptake were noted between the 1.5- and 0.5-mg groups. Although there were differences in uptake over time, and in the 1.5- versus 0.5-mg cohorts, these differences were fairly small, suggesting that ^89^Zr-Df-IAB22M2C will provide a relatively stable signal despite variability in uptake time and minibody mass doses that can occur during clinical studies.

The radiation exposure for ^89^Zr-Df-IAB22M2C, with an effective dose (ICRP 103 (*18*)) of 0.65 ± 0.080 mSv/MBq, was comparable to that for other ^89^Zr-labeled imaging probes (19–23). The relative organ doses from ^89^Zr-Df-IAB22M2C were also comparable to other ^89^Zr-labeled imaging probes, although the spleen dose for ^89^Zr-Df-IAB22M2C was higher. Comparison of groups in the dose-expansion cohort revealed similar dosimetry in subjects who received 1.5 mg of minibody compared with 0.5 mg, with a trend toward lower absorbed doses in the spleen (11 vs. 15 mGy/MBq, respectively) and bone marrow (0.68 vs. 0.81 mGy/MBq, respectively) and a lower effective dose (0.64 vs. 0.67 mSv/MBq, respectively) at the higher mass dose.

Analysis of ^89^Zr-Df-IAB22M2C uptake in tumor lesions revealed maximum uptake at 24–48 h after injection, with slightly higher uptake in the 1.5-mg cohort than in the 0.5-mg cohort, similar to CD8-rich tissues. Although the number of patients was small, most (88%) tumor lesions were radiotracer-avid in patients on immunotherapy, which may reflect the modulation of the immune system and infiltration of tumor lesions by CD8+ leukocytes. A variety of different lesions (lung nodules, nodal metastases, liver metastases), including large lesions, had radiotracer activity at background, demonstrating that ^89^Zr-Df-IAB22M2C has low nonspecific uptake and thus has the potential to quantify CD8+ leukocytes across a wide dynamic range, including those with few to no CD8+ cells, often termed “immune desert” on histologic appearance (24). Although this trial was not designed to correlate tumor uptake with response to therapy, clinical follow-up was available for 3 patients with metastatic melanoma or hepatocellular carcinoma on immunotherapy (pembrolizumab or nivolumab). All 3 patients demonstrated increased ^89^Zr-Df-IAB22M2C uptake in tumor lesions after initiation of immunotherapy, indicating the presence of CD8+ tumor–infiltrating leukocytes, and correlated with subsequent benefit from immunotherapy. Interestingly, all 3 patients had variable uptake at sites of metastases (Supplemental Table 2), with some lesions demonstrating marked uptake (SUV_MAX_ ≥ 10) and other lesions near background activity, suggesting that the kinetics of response might vary between lesions and the presence of one or more PET-positive lesions might be enough to predict response. Although formal study in larger cohorts is needed, these cases illustrate the potential CD8 PET/CT imaging could ultimately have in clinical care to help assess response to immunotherapy.

^18^F-FDG and ^18^F-FLT PET/CT have also been used to assess response to immunotherapy (25–32). However, these probes do not specifically target the immune system, so changes in organ and tumor uptake can be difficult to interpret. Recently, the results from a PET imaging trial with ^89^ZED88082A, a CD8-targeted probe, were presented (33). ^89^ZED88082A demonstrated uptake in the spleen, lymph nodes, and bone marrow similar to that of ^89^Zr-Df-IAB22M2C; however, comparison of tumor uptake is difficult given differences in patient populations.

One limitation of this study is the heterogeneous, small patient population, with different tumor types, tumor burden, and treatment history. However, despite these differences the scans were remarkably similar, with comparable normal-tissue biodistribution and stable uptake in both CD8-rich (SUV_MAX_ range, 3.7–58) and CD8-poor (SUV_MAX_ range, 0.35–0.60) tissues (based on known histology of these tissues rather than directly on biopsy material from study patients) from 24 h onward. An additional limitation of this study is a lack of correlative biopsy data, although the biodistribution of ^89^Zr-Df-IAB22M2C aligned with the expected distribution of CD8+ leukocytes, with saturable signal in CD8-rich tissues at higher doses of cold minibody. An ongoing phase 2 trial (NCT03802123) will test both the diagnostic performance and the predictive performance of ^89^Zr-Df-IAB22M2C, by correlating CD8 signal on PET/CT imaging to CD8+ T-cell infiltration from biopsy samples, and response to cancer immunotherapy, respectively.

## CONCLUSION

This first-in-humans study demonstrated that PET imaging with ^89^Zr-Df-IAB22M2C is safe and well tolerated, and has the potential to visualize the whole-body biodistribution of CD8+ leukocytes in tumors and reference tissues, which may predict response to immunotherapy. The results from this study, including the optimal scan timing (24 h after injection) and minibody mass dose (1.5 mg), are being used in the phase 2 study of ^89^Zr-Df-IAB22M2C, which is currently under way.

## DISCLOSURE

This research was supported by ImaginAb, Inc., the Parker Institute for Cancer Immunotherapy, and the Radiochemistry & Molecular Imaging Probe Core of Memorial Sloan Kettering Cancer Center, supported by NIH/NCI Cancer Center support grant P30 CA008748. Michael D. Farwell receives consulting fees and grant/research support from ImaginAb. Matthew D. Hellmann has equity in Shattuck Labs, Immunai, and Arcus, and he has a patent filed by his institution related to the use of tumor mutation burden to predict response to immunotherapy (PCT/US2015/062208), which has received licensing fees from PGDx. Ron Korn serves as CMIO of ImaginAb. Alessandro Mascioni is employed by ImaginAb. William Le serves as VP of Operations at ImaginAb. Ian Wilson serves as CEO of ImaginAb. Michael S. Gordon receives consulting fees from ImaginAb and Imaging Endpoints. Anna M. Wu receives consulting fees from ImaginAb. Gary A. Ulaner receives consulting fees from ImaginAb and is a member of the Scientific Advisory Board for ImaginAb. Jedd D. Wolchock has equity in Tizona Pharmaceuticals, Adaptive Biotechnologies, Imvaq, Beigene, Linneaus, Apricity, Arsenal IO, and Georgiamune. Michael A. Postow receives grant/research support from ImaginAb. Neeta Pandit-Taskar has served as a consultant or been on an advisory board for and has received honoraria from ImaginAb, and receives grant/research support from ImaginAb. No other potential conflict of interest relevant to this article was reported.

## Notes

KEY POINTS **QUESTION:** Is it feasible to image CD8+ leukocytes in patients with cancer using ^89^Zr-IAB22M2C PET/CT? **PERTINENT FINDINGS:** ^89^Zr-Df-IAB22M2C was found to be safe and well tolerated, with tumor uptake spanning a wide dynamic range. Additionally, the optimal scan timing (24 h after injection) and minibody mass dose (1.5 mg) were selected. In 3 cases with clinical follow-up, increased ^89^Zr-Df-IAB22M2C uptake in tumor lesions correlated with response. **IMPLICATIONS FOR PATIENT CARE:** CD8 PET/CT imaging with ^89^Zr-Df-IAB22M2C is currently being studied as a predictor of or early measure of response to cancer immunotherapy.
